# Efficient and Low Cytotoxicity Gene Carriers Based on Amine-Functionalized Polyvinylpyrrolidone

**DOI:** 10.3390/polym12112724

**Published:** 2020-11-17

**Authors:** Anselmo Del Prado, Ana Civantos, Enrique Martínez-Campos, Pavel A. Levkin, Helmut Reinecke, Alberto Gallardo, Carlos Elvira

**Affiliations:** 1 Instituto de Ciencia y Tecnología de Polímeros, CSIC, Juan de la Cierva 3, 28006 Madrid, Spain; anitacivantos@gmail.com (A.C.); e.martinez.campos@ictp.csic.es (E.M.-C.); hreinecke@ictp.csic.es (H.R.); gallardo@ictp.csic.es (A.G.); celvira@ictp.csic.es (C.E.); 2 Departamento de Química Orgánica, Facultad de Ciencias, Universidad Autónoma de Madrid, 28049 Madrid, Spain; 3 Institute of Biofunctional Studies (IEB), Tissue Engineering Group, (UCM), Associated Unit to the Institute of Polymer Science and Technology (ICTP-CSIC), Paseo de Juan XXIII 1, 28040 Madrid, Spain; 4 Institute of Biological and Chemical Systems—Functional Molecular Systems (IBCS-FMS), Karlsruhe Institute of Technology (KIT), 76344 Eggenstein-Leopoldshafen, Germany; pavel.levkin@kit.edu

**Keywords:** polyvinylpyrrolidone, cationic polymers, biomaterials, cytocompatibility, transfection

## Abstract

Non-viral vectors are a safety tool for gene therapy to deliver therapeutic genes. Among the different non-viral vectors, polyvinylpyrrolidone (PVP), a well-known hydrosoluble, neutral, and non-toxic polymer, satisfies the requirements and becomes a suitable candidate for gene delivery. In this study, we describe the preparation of polyvinylpyrrolidones decorated with pyrrolidine, piperidine, and piperazine groups, and evaluate them in vitro as non-viral gene carriers. The properties of these new systems are compared with those of hyperbranched polyethyleneimine (PEI) used as a positive control. Their ability to complex DNA at different N/P molar ratios, from 1:1 up to 10:1, was studied through agarose gel electrophoresis and dynamic light scattering. The resulting complexes (polyplexes) were characterized and evaluated in vitro with murine fibroblast (Swiss 3T3) as non-viral gene carriers, using luciferase as the reporter gene and a calcein cytocompatibility assay. All the copolymers condensed DNA to a particle average size between 100–400 nm when used at N/P ratios of 4:1 or higher. The copolymers with piperidine groups showed higher transfection efficiency than the pyrrolidine and piperazine modified copolymers, and even higher than the positive control of PEI at N/P ratios of 4:1 or higher. All the synthesized polyplexes from an aminated PVP displayed a general tendency of high cytocompatibility (75–95%) in comparison with the positive control PEI (55%).

## 1. Introduction

Gene therapy, considered a promising approach in the biomedical field, seeks the delivery of therapeutic genes into cells or tissues to treat and prevent acquired and inherited diseases, which are associated to altered or deficit genetic expression [1,2]. Gene therapy begins with the identification of the responsible gene that generates the disease, followed by the production of its identical healthy clone, also referred to as the therapeutic gene. This therapeutic material needs a suitable vehicle to protect the plasmid and avoid degradation in physiological media, as well as express the therapeutic protein by transfection in the nucleus.

The use of the correct vector to deliver the therapeutic gene is a key point in successful gene therapy [3]. Two main groups of vectors have been identified: Viral and non-viral vectors. In the early 1990s, viral carriers were used in clinical trials due to their high transfection efficiency. However, strong safety concerns emerged which limited their use in human gene therapy. These safety limitations were associated with the type of viral carrier (e.g., retroviruses, adenoviruses, and adeno-related viruses), which triggered acute toxicity, inflammatory effects, and insertional mutagenesis [4,5,6]. In contrast, the use of non-viral vectors has drawn significant attention in the research community due to their low immunogenicity, relatively low production cost, high biosecurity, and reproducibility. These non-viral carriers solve the safety concerns mentioned above. However, their main drawback lies in its lower transfection efficiency compared to viral vectors. This poor transfection efficiency arises from the type of gene carrier, and the multiple cellular barriers that non-viral vectors found during the gene delivery process. Therefore, the knowledge of the mechanisms of gene delivery and the interaction between non-viral carriers and the lipidic bilayer of the cell membrane has been key to design non-viral vectors capable of overcoming the low genetic transfection [7].

In recent years, a great variety of non-viral vectors have been tailored including polymer-based, lipid-based, peptide-based, and DNA-based materials to offer an alternative medical treatment for tumors [8,9] and brain diseases [10]. Among the different types of non-viral vectors, polymers present feasible synthesis and great versatility in their preparation by a previous design of their structure, morphology, and functionalization. Polymers bearing cationizable (or cationic) groups form polyelectrolyte complexes (polyplexes) with the negative charge of nucleic acids. Most of the polymers used as non-viral vectors are polyamines that contain secondary and tertiary amine groups, which are total or partially protonated under physiological conditions developing electrostatic interactions with the DNA. In addition, the nature of these amines plays an essential role to complex the genetic material, as well as its transport along the cells, release, and transfection at the nucleus. Therefore, there are numerous studies based on the design of aminated polymers for this purpose [11]. Some of the most representative synthetic and natural polymers in this field to date, as well as their innumerable derivatives are poly-L-lysine (PLL) [12,13], poly(α-(4-aminobutyl)-L-glycolic acid) (PAGA) [14], poly(lactide-co-glycolide) (PLGA) [15], polyethyleneimine (PEI) [16,17,18], polyphosphoesters (PPE) [19], polyamidoamine (PAMAM) [20], dextran [10], silk [21], alginate [22], or chitosan [23,24].

Traditionally, PEI has been considered the gold standard for polymer-mediated gene delivery. However, this transfection activity, described as “proton sponge” depends on its molecular weight, length, and morphologies (branched and linear), and is accompanied by a certain cytotoxicity effect [25,26]. For that reason, not only these aminated polymers in their native form have been studied for years, but also the possibility of improving their transfection efficacy and cytocompatibility behavior by modifying their structures remains a challenge. A common example is branched PEI, whose transfection rate is much higher than that of most other cationic polymers, although the high density of amines in its structure provides toxicity. For this reason, functionalization studies of branched PEI have been carried out using groups that increase its biocompatibility, such as polyethylene glycol (PEG) chains [27]. The opposite effect occurs with PLL, whose toxicity is low, but its transfection efficiency is poor. Some studies have reported higher effectiveness through polymer modification, which consisted of the introduction of secondary and tertiary amines to enhance its buffering capacity [28], thus improving its properties when releasing the genetic material into the cytoplasm. Furthermore, more complex entities such as cyclodextrins have been anchored to different polymers to increase the complexing capacity of the genetic material [29,30,31].

A well-known and useful polymer in biomedical applications is polyvinylpyrrolidone (PVP), which is stable, non-toxic, and water soluble. It has shown good properties as an excipient in the DNA administration [32], as support to prepare polymer-drug conjugates [33], and it has even been evaluated as a plasmid vector, for example, by intramuscular injection for the suppression of neovascularization [34], or in the release of angiogenic genes for metastasis suppression [35]. Furthermore, PVP has been attached to other active cationizable components to modulate toxicity, giving as a result graft copolymers with PEI [36], and with dimethylaminoethyl methacrylate (DMAEMA) [37]. Therefore, new derivatives of PVP combine on a common PVP backbone, its non-toxicity, excellent biocompatibility and hydrophilicity, with the complexation capability of the cationizable amino group.

In this work, we describe the application of aminated polymers with a PVP backbone as gene carriers. PVP polymers were functionalized with three different types of amine: Pyrrolidine, piperidine, and piperazine, which have been already used to prepare efficient aminated polymers for DNA release [38,39,40,41,42]. Their application as non-viral gene carriers is studied and the effect of the nature of the amine in the transfection efficiency and cytotoxicity in PVP polymers is compared. The prepared cationizable copolymers have been characterized and evaluated as non-viral vectors in transfection studies in fibroblast 3T3 cells, as well as their biocompatibility.

## 2. Materials and Methods

N-vinylpyrrolidone (VP) from Sigma-Aldrich (Taufkirchen, Germany) was distilled at low pressure and stored at 4 °C. The thermal initiator N,N′-azobisisobutyronitrile (AIBN) from Merck (Darmstadt, Germany) was recrystallized twice in ethanol. Hyperbranched polyethyleneimine (PEI, M_w_ = 25 kDa, *Đ* = 2.5) was purchased from Sigma–Aldrich (Taufkirchen, Germany). All the solvents were used without further purification. The aminated monomers were synthesized following the protocols described elsewhere [43].

The *Gaussia princeps* luciferase plasmid pCMV-GLuc of 6 kpb (New England Biolabs, Ipswich, MA, USA) was used in the transfection assays, whose activity was then evaluated using the Biolux^®^ Gaussia Luciferase Assay Kit (New England Biolabs, Ipswich, MA, USA). Calcein AM (Molecular Probes, Waltham, MA, USA) was used to evaluate the cytotoxicity by inverted fluorescence microscopy (Olympus IX51, Tokyo, Japan).

### 2.1. Synthesis of the Polyvinylpyrrolidone Backbone Polymers

All polymers were synthesized using a conventional free radical polymerization procedure: Monomers and the initiator (AIBN) were dissolved in 1,4-dioxane at concentrations of 1.0 and 1.5 × 10^−2^ M, respectively. Gaseous N_2_ was flushed through the solutions for 20 min. The polymerization reactions were carried out at 60 °C during 24 h to attain total conversion. The homopolymers of the aminated VPs were synthesized, as well as the corresponding copolymers with VP using four different monomer feed ratios, as reported [43]. The number-average molecular weight (*M*_n_) and polydispersity index (*Ð*) of the polymers were measured by gel permeation chromatography (GPC) with a Perkin Elmer (Waltham, MA, USA) chromatographic system equipped with a Waters (Milford, MA, USA) model 2414 refractive index detector, using Styragel (300 × 7.8 mm, 5 μm nominal particle size) HR3 and HR5 water columns. Dimethylformamide (DMF) with 1 wt % LiBr was used as eluent. Measurements were performed at 70 °C at a flow rate of 0.7 mL/min using a polymer concentration of 4 mg/mL. The calibration was performed with monodispersed polystyrene standards in the range of 2.0 and 9000.0 kDa. The resulting data is quoted in Table 1.

### 2.2. Preparation and Characterization of Polymer/Plasmid Complexes

The polymer/plasmid complexes (polyplexes) were prepared in distilled water using various N/P molar ratios (amine from polymer/phosphate from plasmid), ranging from 1:1 to 10:1 of incubation, for 30 min at room temperature in a concentration of 1 µg plasmid in 20 µL water. Polyplexes obtained from commercial PEI were also analyzed in the same ratios and conditions as control patterns.

A 0.9% (*w*/*v*) agarose gel was used in order to assess the polyplex formation, applying 100 V for 30 min. Free plasmid was used as a control. The electrophoresis was carried out in a Tris/Acetate/EDTA buffer (TAE, pH 8) following the standard procedure. Afterward, UV light was used to visualize the plasmid band, marked with ethidium bromide (Sigma-Aldrich, Taufkirchen, Germany).

Polyplexes were sized using dynamic light scattering (NanoZS Malvern Instruments, Malvern, UK), and the ζ potential was analyzed by electrophoretic light scattering using a Zetamaster system (Malvern Instruments, Malvern, UK). Polyplexes were made up using 10 μg of the plasmid and the corresponding amount of polymer per 1 mL of water. An amount of 0.1 mL of the resulting solution was used to carry out the size measurements, and then diluted to 1 mL for the charge surface analyses. Three readings were obtained per sample, at 25 °C allowing 1 min of equilibration time before acquiring the data. The ALV-Correlator Control Software was used for data acquisition using a counting time from 300 to 600 s for each sample.

### 2.3. In Vitro Cellular Studies

For this study, mouse Swiss 3T3 fibroblasts were purchased from the American Type Culture collection (ATCC^®^ CCL-92) (Manassas, VA, USA). Cells were routinely grown in a complete medium composed by Dulbecco’s Modified Eagle Medium (DMEM) (Gibco, Waltham, MA, USA), supplemented with a 10% fetal bovine serum (FBS) and 1% of penicillin/streptomycin (P/S), maintained at 37 °C in a humidified 5% CO_2_ atmosphere.

Transfection experiments were performed using the luciferase gene contained in the pCMV-GLuc plasmid as a reporter gene. An amount of 5000 cells/cm^2^ were seeded in 96-well plates. After 24 h of incubation, the culture medium of each well was replaced by 80 μL of serum-free DMEM and 20 μL of the polyplex solution (1 μg of plasmid and the corresponding of the polymer per well), in which the cells were cultured for 4 h at 37 °C in a humidified 5% CO_2_ atmosphere. Afterward, the medium was replaced by a fresh complete culture medium in which the cells were incubated for an additional 48 h. Finally, the medium from each well was collected and analyzed for the production of luciferase protein, using the commercial kit Biolux^®^ Gaussia Luciferase Assay Kit (New England Biolabs, Ipswich, MA, USA) following the supplier’s protocol. The luciferase expression was quantified by measuring the luminescence as relative luminescence units (RLUs), with a plate reader BioTek Synergy HT (Biotek Instruments, Winooski, VT, USA). All the measurements were taken in triplicate and the cells treated with naked plasmid were used as negative controls.

After performing the transfection measurements, the cells were used to carry out the cell viability assay with calcein AM (Molecular Probes, Waltham, MA, USA), a specific dye that marks alive cells by inverted fluorescence microscopy, and is quantified with a microplate reader BioTek Synergy HT (Biotek Instruments, Winooski, VT, USA). For that, 100 µL of the calcein solution (4 µg/mL) was added to each well. After incubation during 30 min in the same conditions as used in the transfection assay, fluorescence (λ_ex_ 495 nm, λ_em_ 515 nm) was measured with a plate reader BioTek Synergy HT (Biotek Instruments, Winooski, VT, USA), with the data normalized with respect to those of the non-treated cells (100% of viability), and expressed as relative fluorescence values (RFVs). In addition, a qualitative comparison of fluorescence between wells was performed using the inverted fluorescence microscope Olympus IX51 with a filter FITC (λ_ex_ 496 nm, λ_em_ 519 nm) using the analysis software CellD (Olympus, Tokyo, Japan).

All the in vitro experiments were performed in triplicate and an average was taken for analysis. The analysis of variance (ANOVA) was performed using the Graph Path Prims 6 software. The results were statistically analyzed by the application of two-way ANOVA and a value of *p* < 0.05 was considered significant.

## 3. Results and Discussion

**Figure 1:**
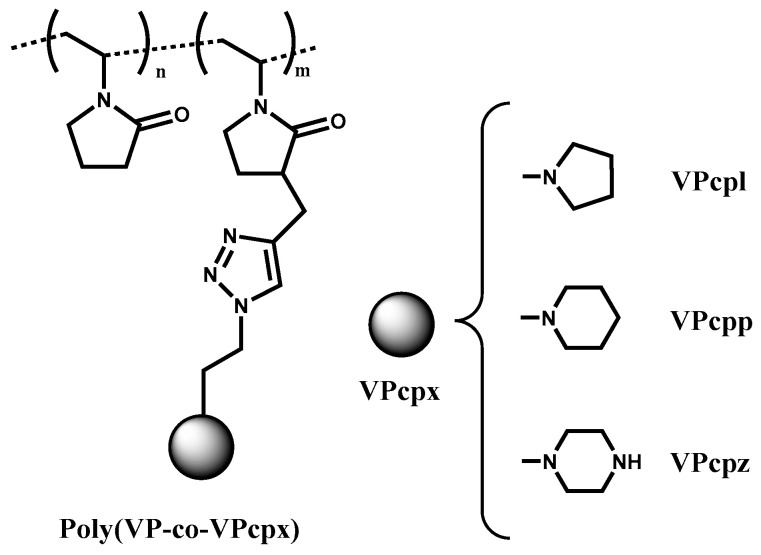
Structure of the evaluated poly(VP-co-VPcpx) copolymers.

A total of fifteen polymers (see Table 1) with a PVP backbone were synthesized and characterized as reported [43], and evaluated as gene carriers in the present work, carrying three different types of amines as side groups in various mol% of amine content (20, 40, 60, 80, and 100). The copolymer systems have been named as poly(VP-co-VPcpx)-Y in order to facilitate the reading, where Y indicates the mol% of amine content in the polymer (20, 40, 60, or 80), and VPx represents the nature of the amine where x may be l (pyrrolidine), p (piperidine), or z (piperazine) (Figure 1). The corresponding homopolymers (aminated monomer only) were denoted as poly(VPcpx), where x is l, p or z, as appropriate. Figure 1 shows the structure of the synthesized copolymers, and Table 1 indicates the monomer feed ratio (*F_VPcpx_*), the monomer composition (*f_VPcpx_*), the molecular weight (*M_n_*), and the polydispersity index (*Ð*).

### 3.1. Polyplexes Analysis

The DNA complexation capacity of each polymer was evaluated by agarose gel electrophoresis (see Figures S1.1–S1.3 in Supplementary Materials), using different N/P molar ratios to achieve the mentioned polyplexes.

Figure S1.1 shows the electrophoretic mobility of the free plasmid pCMV-GLuc (M) versus the polyplexes obtained from poly(VP-co-VPcpl) and the plasmid at different N/P ratios (from 1:1 to 10:1), where the positive control of the PEI and plasmid is shown as ‘C’ in a N/P ratio of 8:1. Essentially, the total complexation is achieved in all the ratios independently of the amino functionalization degree. The resulting agarose gels for the polyplexes prepared from the plasmid and the polymers carrying either piperidine or piperazine are shown in Figures S1.2 and S1.3 (Supplementary Materials), respectively. The complexation using the PVP with piperidine groups was effective for all the N/P ranges used in the preparation of the polyplexes. In the case of polyplexes formed by piperazine functionalized polymers, a less amount of the plasmid was needed in all the polyplexes due to the presence of the double amount of nitrogen per mol of aminated VP monomer (the piperazine ring contains secondary and tertiary amines). The efficiency to compensate for the negative charge of the DNA was lower than for the rest of the polymers, as shown in Figures S1.1–S1.3 of the Supplementary Materials. The plasmid was completely complexed for N/P ratios equal or above to 4:1 in all the polymers, yielding an effective DNA complexation of N/P 2:1 only in those polymers with a 60% and 80% of functionalization. This result indicates that the efficiency to complex the plasmid was not enough for the piperazine monomer probably due to the proximity of the amine groups, which makes it unlikely to achieve both positive charges and polymer conformations appropriate for DNA complexation.

**Figure 2:**
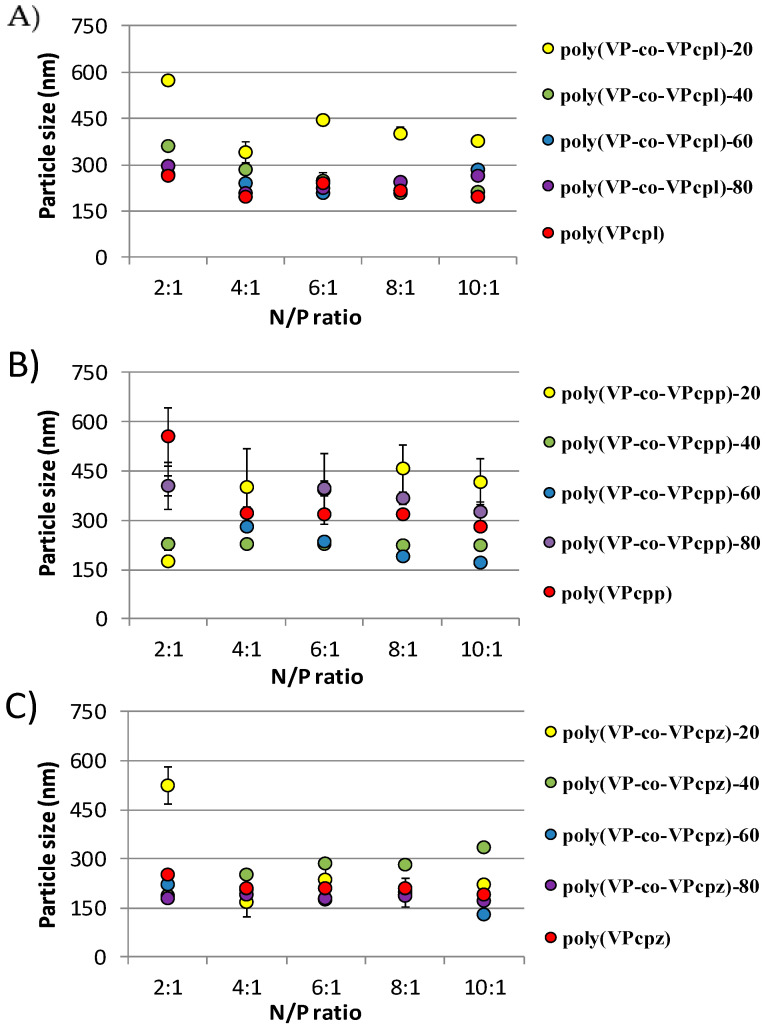
Average particle size measurements of the polymer/DNA complexes derived from (**A**) poly(VP-co-VPcpl), (**B**) poly(VP-co-VPcpp), and (**C**) poly(VP-co-VPcpz) copolymers measured at different N/P ratios in MilliQ water.

**Figure 3:**
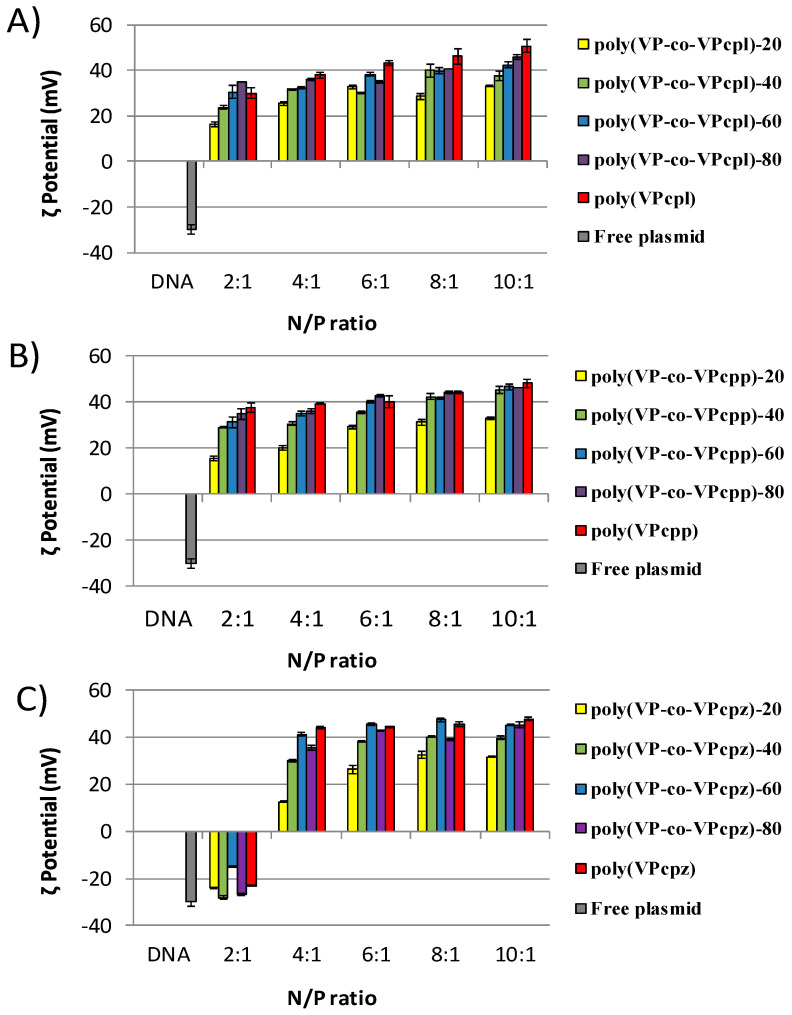
*ζ* potential measurements of the polymer/DNA complexes derived from (**A**) poly(VP-co-VPcpl), (**B**) poly(VP-co-VPcpp), and (**C**) poly(VP-co-VPcpz) copolymers measured at different N/P ratios in MilliQ water. Free plasmid is represented as a reference.

The size and ζ potential of the polyplexes were measured by dynamic light scattering (DLS), represented in Figure 2 and Figure 3, respectively. The analysis of the size of the prepared polyplexes is shown in Figure 2, obtaining a range of 200–500 nm for those prepared by polymers carrying pyrrolidine (A) and piperidine (B), and a range of 100–300 nm for those prepared by polymers carrying piperazine (C). It should be noted that, comparing globally all the measures, the polymers with piperazine groups yielded smaller polyplexes than the others carrying only tertiary amines. As mentioned before, the poor DNA complexation capacity of the piperazine decorated polymers may be the consequence of the lower particle size. On the other hand, generally the particle size is bigger at a N/P ratio of 2:1 probably due to the lower charge density and also to the corresponding lower electrostatic repulsion, which allows for the formation of bigger aggregates and the particle sizes increase. These results agree with the resulting lower superficial charge density of the polyplexes of the N/P ratio 2:1 in comparison with the others, as mentioned below.

Figure 3 shows the values of the ζ potential for all the polyplexes, displaying a positive charge in all the cases, except for those obtained from polymers carrying piperazine groups with a N/P ratio of 2:1, which displayed a negative charge (between −15 and −25 mV). This is in agreement with the results obtained from the electrophoresis previously discussed, and the mentioned inefficiency to complex both amines of the piperazine group. The other complexes showed a tendency that depends on the composition: The higher the amine content in the polymer, the higher the ζ potential. With the exception of the complexes prepared from polymers with 20% of amine content and N/P ratios lower than 6:1, all the polyplexes displayed values of the ζ potential close to or higher than +30 mV, which is considered the threshold for particles with a high stability in the solution (low aggregation).

### 3.2. Cell Culture Studies

Mouse Swiss 3T3 fibroblasts were seeded in a 96-well plate and incubated in a complete medium during 24 h before the addition of the corresponding polyplexes. After the incubation of the polyplexes in the absence of the serum, the media was changed for a complete medium, and incubated for an additional 48 h. After that, a luminescence analysis was carried out to determine the transfection efficiency of the polymers used in the preparation of the polyplexes. In this study, naked plasmid was also used as a negative control in which no transfection will occur, similar to the cells without any treatment. Polyplexes in N/P ratios from 2:1 to 10:1 were used in these studies, excluding polyplexes formed in a 1:1 N/P ratio due to their low DNA complexation capacity (see Figures S1.1–S1.3 in Supplementary Materials). In addition, PEI polyplexes were used as positive controls in the same N/P ratios than those of the evaluated polymers. 

**Figure 4:**
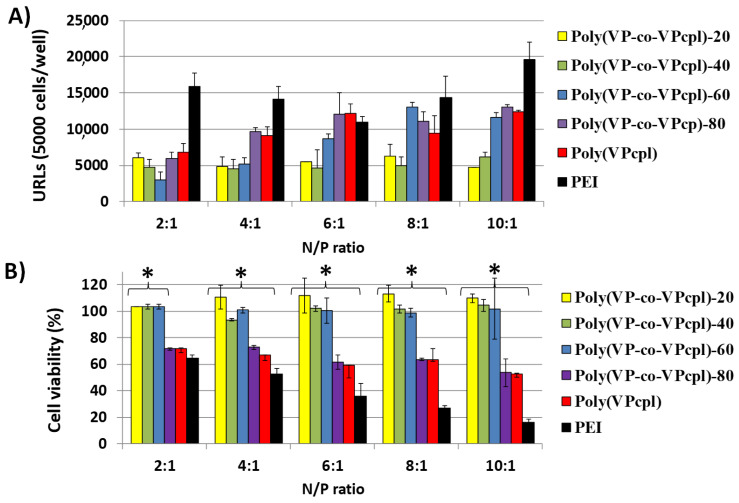
(**A**) Transfection efficiency in Swiss 3T3 cells of polyplexes prepared from poly(VP-co-VPcpl) and GLuc plasmid at different N/P ratios (polymer/DNA) expressed as relative luminescence units (URLs), and (**B**) cytocompatibility (% cellular viability), obtained with the calcein AM assay. Results were normalized to the levels of non-treated cells as relative fluorescence values (FRVs) after the transfection assay. * Denotes a significant difference with the positive control PEI at the same N/P ratio (one-way ANOVA, *p* < 0.05).

**Figure 5:**
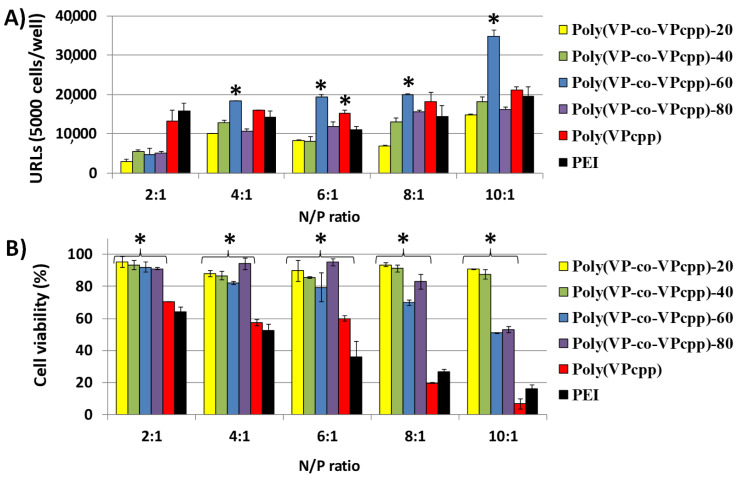
(**A**) Transfection efficiency in Swiss 3T3 cells of the polyplexes prepared from poly(VP-co-VPcpp) and GLuc plasmid at different N/P ratios (polymer/DNA) expressed as relative luminescence units (URLs), and (**B**) cytocompatibility (% cellular viability), obtained with the calcein AM assay. Results were normalized to the levels of the non-treated cells as relative fluorescence values (FRVs) after the transfection assay. * Denotes a significant difference with the positive control PEI at the same N/P ratio (one-way ANOVA, *p* < 0.05).

**Figure 6:**
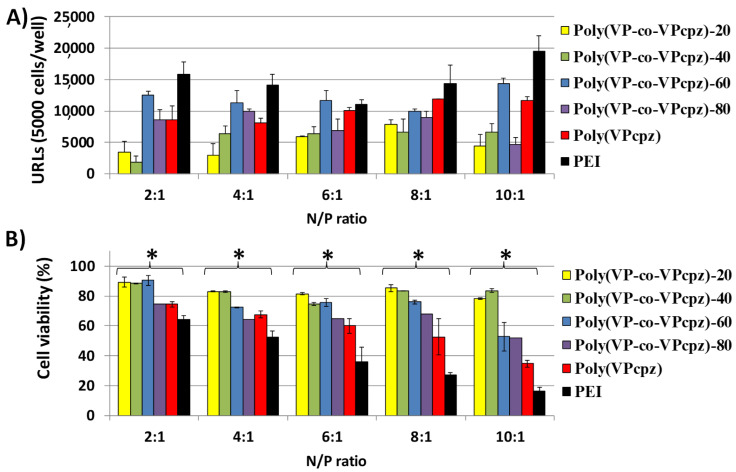
(**A**) Transfection efficiency in Swiss 3T3 cells of the polyplexes prepared from poly(VP-co-VPcpz) and GLuc plasmid at different N/P ratios (polymer/DNA) expressed as relative luminescence units (URLs), and (**B**) cytocompatibility (% cellular viability), obtained with the calcein AM assay. Results were normalized to the levels of the non-treated cells as relative fluorescence values (FRVs) after the transfection assay. * Denotes a significant difference with the positive control PEI at the same N/P ratio (one-way ANOVA, *p* < 0.05).

Figure 4A, Figure 5A and Figure 6A show the obtained relative luminescence units (URLs) for the polyplexes prepared from the polymers functionalized with pyrrolidine, piperidine, and piperazine, respectively. Their toxicity was evaluated by cell viability assays of Swiss 3T3 fibroblasts with calcein after the transfection assays, as described in the experimental part, and the results are shown in Figure 4B, Figure 5B and Figure 6B for those polyplexes prepared from polymers functionalized with pyrrolidine, piperidine, and piperazine, respectively.

Polyplexes with pyrrolidine as pendant groups gave generally a lower transfection efficiency than the positive control (hyperbranched PEI). As shown in a comparative manner in Figure 4A, the higher the amount of pyrrolidine groups in the polymer, the higher the transfection efficiency. It should be noted that for those polyplexes carrying 60% or less of the amount of pyrrolidine, the cell viability was practically 100% (Figure 4B), being significantly less toxic (*p* < 0.05) than PEI. However, a low transfection efficiency was obtained for polyplexes formed with poly(VP-coVPcpl)-20 and poly(VP-coVPcpl)-40 when compared with PEI. In polyplexes with polymers with a higher presence of pyrrolidine (80% and 100%), the cell viability decreased in comparison with those polyplexes with a lower amount of pyrrolidine. However, the systems were significantly less toxic than PEI in N/P ratios of 4:1 and higher.

The resulting transfection efficiency of the polyplexes obtained from polymers carrying piperidine was comparatively better than that obtained for the pyrrolidine set, with similar efficiency as the PEI control, and even statistically better in the case of those polyplexes prepared from poly(VP-co-VPcpp)-60 (Figure 5A). In addition, polyplexes prepared with 80% or less of the amount of piperidine were significantly more cytocompatible than the PEI control. The cell viability of polyplexes from the homopolymer was comparatively similar to that of PEI, decreasing down to 10% with a N/P ratio of 10:1 (Figure 5B).

The corresponding polyplexes prepared from the piperazine-modified PVPs behaved similarly to those of the pyrrolidone system as their transfection efficiencies were similar and never higher than those observed for the PEI control. Only the poly(VP-co-VPcpz)-60 polyplexes behaved in a similar way to PEI when used in all the range of N/P ratios, and some of them when used in a N/P ratio of 6:1 or lower (Figure 6A). In addition, the cell viability analysis shows that piperazine amounts of 60% or less reached low toxicity in most of the cases, being generally more biocompatible than PEI (Figure 6B). As a peculiarity, a positive but not high transfection efficiency was achieved with these polyplexes at a N/P ratio of 2:1, which showed a negative ζ potential. This may be a consequence of an ineffective DNA complexation as the piperazine group has two amine sites in the same ring, which provokes positive charge repulsions and affects the DNA-polymer interaction, as mentioned before. Regarding cell viability, all the piperazine set of polyplexes was significantly less toxic than the PEI control, especially those with less VPcpz content as expected. Nevertheless, copolymers with a low piperazine content (20–60%) resulted as more cytotoxic (80% of viability) than the pyrrolidine and piperidine sets (80–100% of viability) with the same amine content, and always less toxic than the PEI control.

**Figure 7:**
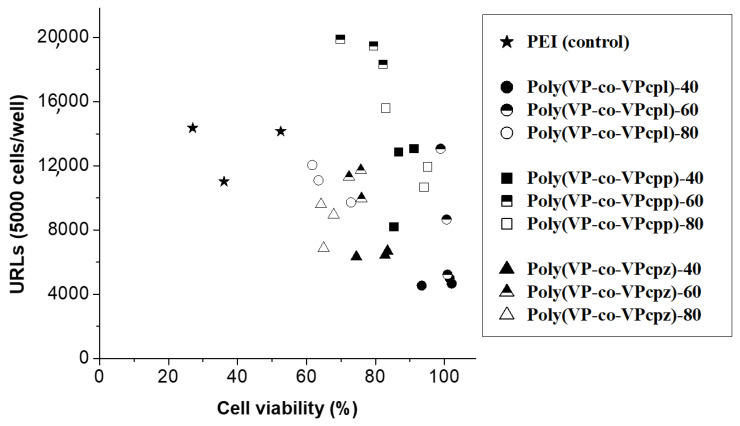
Comparison of the transfection efficiency in relative luminescence units (URLs) of the copolymers carrying pyrrolidine (circle), piperidine (square), or piperizine (triangle) with different amine contents (40%-filled, 60%-half filled, and 80%-empty) versus their cell viability of the analyzed Swiss 3T3 fibroblasts at N/P ratios of 4:1, 6:1, and 8:1. The results obtained from the PEI control in the same condition are plotted as stars.

Figure 7 summarizes the results of the transfection efficiency and cell viability assays of the mentioned polyplexes with fibroblast Swiss 3T3 cells, using the aminated PVPs with 40% (filled), 60% (half filled), and 80% (empty) of pyrrolidine (circle), piperidine (square), or piperazine (triangle) content in N/P ratios of 4:1, 6:1, and 8:1. The same experiments for PEI are plotted as a reference (star). The cell viability of the aminated PVPs was always higher than 60%, in contrast to PEI polyplexes that never get over 60%. Generally, polymers with higher cytotoxicity were those with higher amine content, being close to the 60% of cell viability, except polymers carrying pyrrolidine groups which exhibited a cell viability of 85–95%. Polymers that showed a transfection efficiency lower than 10.000 URLs were those with 40% of amine content, being equally low cytotoxic. Polymers carrying piperidine groups showed a similar transfection efficiency than the PEI controls, but were significantly more cytocompatible (higher than 75%). Furthermore, poly(VP-coVPcpp)-60 showed a transfection efficiency significantly higher (*p* < 0.05) than PEI.

Comparing the three types of polymers, the cell viability studies have demonstrated the effect of the presence of VP and the PVP-type skeleton, where copolymers, and in some cases the homopolymers, were more cytocompatible than the PEI control. The higher the VP composition, the lower the cytotoxicity, being fully cytocompatible even with 20% and 40% of amine content. It is noteworthy to mention that many of these high cytocompatibility sets showed transfection efficiencies statistically similar to those of PEI, highlighting the piperidine system that appears to be slightly more effective than the pyrrolidine and piperazine sets (see Figure 7).

In short, based on the results shown above, the intermediate compositional range for the three sets of copolymers (40–60%, and even in the case of piperidine 80%) presented an excellent transfection efficiency/cytocompatibility balance, better than the PEI control. In this VP/VPamine ratio, the VP content and the PVP skeleton, as well as the segmental charge density (lower than that of the homopolymer or the PEI control), makes their polyplexes cytocompatible and efficient in transfection. In addition, the charge density itself and the surface charge had a great influence on the complexation and intracellular release of DNA, as well as on the interaction with the cell membrane and with the cytotoxicity and internalization phenomena. It is worth highlighting the piperazine system in a 2:1 ratio, which even presenting a negative surface charge has shown to be efficient in transfection. The nature of the amine is important since it modulates the interaction with DNA and with the cell membrane, and has an important influence on the exit of the complex from the lysosome (and its participation in the ATPase proton pump). In this sense, it seems that the systems carrying only tertiary amines (pyrrolidine and piperidine) form larger polyplexes and are slightly more cytocompatible than those of secondary amine. However, a correlation of these aspects with the transfection efficiency has not been found.

**Figure 8:**
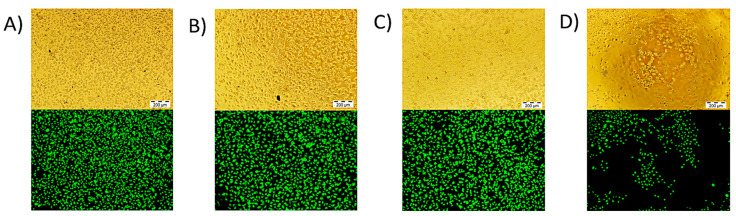
Micrographs obtained after incubation during 20 min of the Swiss 3T3 fibroblasts cells with the calcein AM reagent after the transfection assay with the polyplexes derived from (**A**) poly(VP-co-VPcpl)-60, (**B**) poly(VP-co-VPcpp)-60, (**C**) poly(VP-co-VPcpz)-60, and (**D**) PEI control at a N/P ratio of 8:1 (scale bar: 200 µm).

To confirm the cytotoxicity effect of the polyplexes, a qualitative analysis was performed labelling cells with the calcein AM reagent used after the transfection efficiency and cell viability experiments. Different micrographs were taken in the inverted fluorescence microscope 30 min after the incubation of the Swiss 3T3 fibroblasts with calcein (at 37 °C and 5% CO_2_ in a humid atmosphere) (see Figure 8). The cells incubated with the polyplexes prepared from copolymers with a 60% of amine content of pyrrolidine (A), piperidine (B) and piperazine (C) grew normally, showing a high cell density and it was also observed that most of the cells were kept alive (calcein-labeled fluorescent alive cells) after the transfection assay during 48 h. On the other hand, the cells incubated with the polyplexes obtained from the PEI control (D) showed a lower proliferated-state, denoting higher cytotoxicity, and being able to observe that a large number of the cells analyzed did not emit fluorescence as they were dead. This analysis agrees with the results represented in Figure 7, which clearly shows that cell viability of the polymers derived from PVP was higher than 75%, while the cytotoxicity of the PEI control did not exceed the 55% of the cell viability in any of the cases.

## 4. Conclusions

The functionalization of neutral and biocompatible PVP to cationizable non-viral gene vectors plays an important role on their ability and features as gene delivery carriers. The DNA complexation capacity of the aminated copolymers were fully effective when using N/P ratios of 4:1 or higher, exhibiting polyplexes with an average particle size between 100 and 400 nm, and a ζ potential of a +20 to +40 mV range. These characteristics allowed them to behave as efficient gene carriers in Swiss 3T3 fibroblasts, similar or better than the PEI positive control. The copolymers with piperidine groups showed higher transfection efficiency than the PEI, optimal with a 60% of amine content and 4:1 N/P ratio or higher. Pyrrolidine and piperazine copolymers showed a similar transfection efficiency than PEI when used at 6:1 and 8:1 N/P ratios, but never higher in any case. The cytocompatibility studies have shown that the PVP backbone makes these copolymers suitable to prepare polyplexes highly or fully cytocompatible, superior to the PEI controls, and the VP/VPcpx ratio on the copolymers plays a key factor on the transfection efficiency/cytocompatibility balance.

## Figures and Tables

**Table 1:** Characterization of the poly(VP-co-VPcpx) copolymers.

***F_VPcpl_***	***f_VPcpl_*^a^**	***M_n_*** (kDa) **^b^**	***Đ*^b^**
0.20	0.18	46.2	1.54
0.40	0.43	46.4	1.65
0.60	0.54	44.0	1.75
0.80	0.78	41.9	1.78
1.00	1.00	33.4	1.72
***F_VPcpp_***	***f_VPcpp_*^a^**	***M_n_*** (kDa) **^b^**	***Đ*^b^**
0.20	0.17	42.9	1.46
0.40	0.44	39.2	1.44
0.60	0.60	36.7	1.49
0.80	0.83	28.2	1.52
1.00	1.00	23.2	1.51
*F_VPcpz-Boc_* *	*f_VPcpz-Boc_*^a,^*	***M_n_*** (kDa) ^b,^*	*Đ*^b,^*
0.20	0.19	41.9	2.10
0.40	0.36	42.3	2.15
0.60	0.58	45.7	1.94
0.80	0.78	48.3	1.82
1.00	1.00	63.3	1.49

^a^ Calculated by 1 H NMR. ^b^ Determined by GPC in DMF with a LiBr 1% weight. * Data collected for the poly(VP-co-VPcpz) copolymers in the protected form. Data obtained from Ref. [43].

## Supplementary Materials

The following are available online at https://www.mdpi.com/2073-4360/12/11/2724/s1. Figure S1.1: Electrophoretic mobility of plasmid DNA in the polyplexes derived from poly(VP-co-VPcpl) copolymers studied in this work, at different N/P molar ratios ranging from 1:1 to 10:1, in a TBE buffer. M = free plasmid and C = PEI/plasmid at a N/P ratio of 8:1 as the control; Figure S1.2: Electrophoretic mobility of plasmid DNA in the polyplexes derived from poly(VP-co-VPcpp) copolymers studied in this work, at different N/P molar ratios ranging from 1:1 to 10:1, in a TBE buffer. M = free plasmid and C = PEI/plasmid at a N/P ratio of 8:1 as the control; Figure S1.3: Electrophoretic mobility of plasmid DNA in the polyplexes derived from poly(VP-co-VPcpz) copolymers studied in this work, at different N/P molar ratios ranging from 1:1 to 10:1, in a TBE buffer. M = free plasmid and C = PEI/plasmid at a N/P ratio of 8:1 as the control.
