# Supplementary material for: Efficient and Low Cytotoxicity Gene Carriers Based on Amine-Functionalized Polyvinylpyrrolidone
Source: Polymers (Basel). 2020 Nov 17;12(11):2724. doi: 10.3390/polym12112724 (PMC7698542; doi:10.3390/polym12112724)
Supplement: Supplementary file 1 [file polymers-12-02724-s001.pdf]

*Supplementary Materials*

# Efficient and Low Cytotoxicity Gene Carriers Based on Amine-Functionalized Polyvinylpyrrolidone

**Anselmo Del Prado [1,2,*], Ana Civantos [1,3], Enrique Martínez-Campos [1,3], Pavel A. Levkin [4], Helmut Reinecke [1], Alberto Gallardo [1] and Carlos Elvira [1]**

[1] Instituto de Ciencia y Tecnología de Polímeros, CSIC, Juan de la Cierva 3, 28006, Madrid, Spain; anitacivantos@gmail.com (A.C.); e.martinez.campos@ictp.csic.es (E.M.-C.); hreinecke@ictp.csic.es (H.R.); gallardo@ictp.csic.es (A.G.); celvira@ictp.csic.es (C.E.)

[2] Departamento de Química Orgánica, Facultad de Ciencias, Universidad Autónoma de Madrid, 28049 Madrid, Spain.

[3] Institute of Biofunctional Studies (IEB), Tissue Engineering Group, (UCM), associated unit to the Institute of Polymer Science and Technology (ICTP-CSIC), Paseo de Juan XXIII 1, 28040 Madrid, Spain

[4] Institute of Biological and Chemical Systems – Functional Molecular Systems (IBCS-FMS), Karlsruhe Institute of Technology (KIT), 76344 Eggenstein-Leopoldshafen, Germany; pavel.levkin@kit.edu

* Correspondence: anselmo.delprado@uam.es

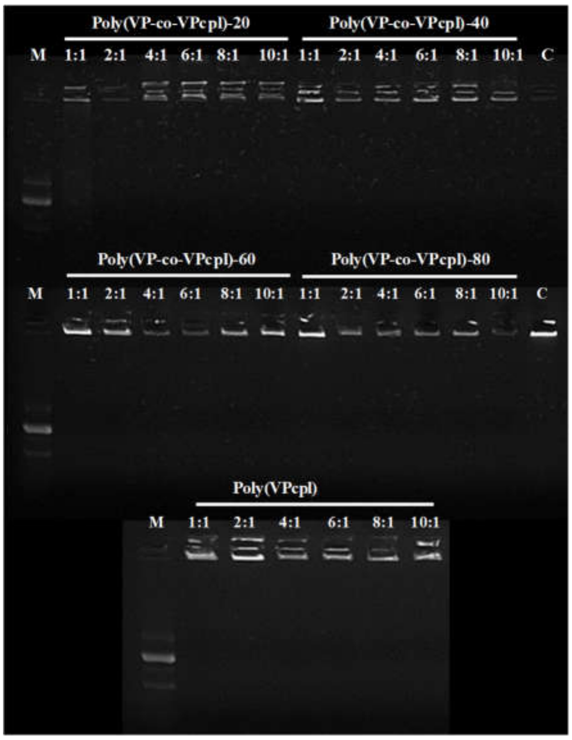


**Figure S1.1.** Electrophoretic mobility of plasmid DNA in the polyplexes derived from poly(VP-co-VPcpl) copolymers studied in this work, at different N/P molar ratios ranging from 1:1 to 10:1, in a TBE buffer. M = free plasmid and C = PEI/plasmid at a N/P ratio of 8:1 as the control.

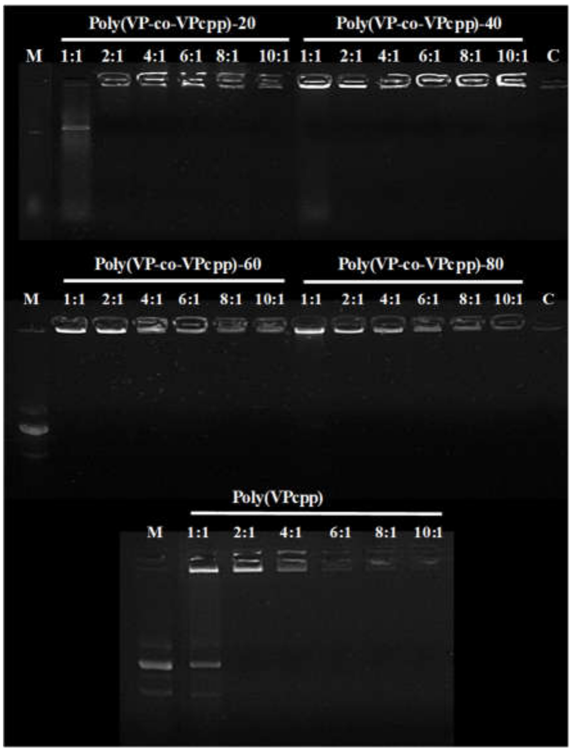


**Figure S1.2.** Electrophoretic mobility of plasmid DNA in the polyplexes derived from poly(VP-co-VPcpp) copolymers studied in this work, at different N/P molar ratios ranging from 1:1 to 10:1, in a TBE buffer. M = free plasmid and C = PEI/plasmid at a N/P ratio of 8:1 as the control.

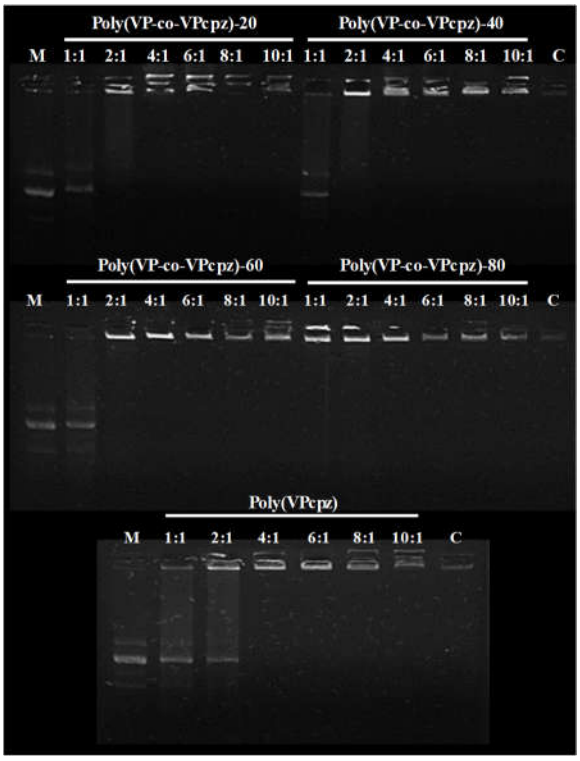


**Figure S1.3.** Electrophoretic mobility of plasmid DNA in the polyplexes derived from poly(VP-co-VPcpz) copolymers studied in this work, at different N/P molar ratios ranging from 1:1 to 10:1, in a TBE buffer. M = free plasmid and C = PEI/plasmid at a N/P ratio of 8:1 as the control.
